# Carbuncles

**Published:** 1901-03

**Authors:** 


### Carbuncles.

Creel has relied on Ecthol given internally, in doses of a teaspoonful, in cases of carbuncle, flaxseed poultices applied locally, emptying of pus, scraping out of dead tissue and cleansing with peroxide of hydrogen; after this a topic application of Ecthol on absorbent cotton every four to eight hours. The average duration of this treatment in his cases was ten days.—*Journal American Medical Association.*

